# Fruit Photosynthesis: More to Know about Where, How and Why

**DOI:** 10.3390/plants12132393

**Published:** 2023-06-21

**Authors:** Andreia Garrido, Artur Conde, João Serôdio, Ric C. H. De Vos, Ana Cunha

**Affiliations:** 1 Centre of Molecular and Environmental Biology (CBMA), Department of Biology, University of Minho, Campus de Gualtar, 4710-057 Braga, Portugalarturconde@bio.uminho.pt (A.C.); 2 Centre for Environmental and Marine Studies (CESAM), Department of Biology, University of Aveiro, Campus de Santiago, 3810-193 Aveiro, Portugal; jserodio@ua.pt; 3 Business Unit Bioscience, Wageningen Plant Research, Wageningen University and Research Centre (Wageningen-UR), P.O. Box 16, 6700 AA Wageningen, The Netherlands; ric.devos@wur.nl

**Keywords:** fruit characteristics, fruit tissues, photosynthetic activity, functions

## Abstract

Not only leaves but also other plant organs and structures typically considered as carbon sinks, including stems, roots, flowers, fruits and seeds, may exhibit photosynthetic activity. There is still a lack of a coherent and systematized body of knowledge and consensus on the role(s) of photosynthesis in these “sink” organs. With regard to fruits, their actual photosynthetic activity is influenced by a range of properties, including fruit anatomy, histology, physiology, development and the surrounding microclimate. At early stages of development fruits generally contain high levels of chlorophylls, a high density of functional stomata and thin cuticles. While some plant species retain functional chloroplasts in their fruits upon subsequent development or ripening, most species undergo a disintegration of the fruit chloroplast grana and reduction in stomata functionality, thus limiting gas exchange. In addition, the increase in fruit volume hinders light penetration and access to CO_2_, also reducing photosynthetic activity. This review aimed to compile information on aspects related to fruit photosynthesis, from fruit characteristics to ecological drivers, and to address the following challenging biological questions: why does a fruit show photosynthetic activity and what could be its functions? Overall, there is a body of evidence to support the hypothesis that photosynthesis in fruits is key to locally providing: ATP and NADPH, which are both fundamental for several demanding biosynthetic pathways (e.g., synthesis of fatty acids); O_2_, to prevent hypoxia in its inner tissues including seeds; and carbon skeletons, which can fuel the biosynthesis of primary and secondary metabolites important for the growth of fruits and for spreading, survival and germination of their seed (e.g., sugars, flavonoids, tannins, lipids). At the same time, both primary and secondary metabolites present in fruits and seeds are key to human life, for instance as sources for nutrition, bioactives, oils and other economically important compounds or components. Understanding the functions of photosynthesis in fruits is pivotal to crop management, providing a rationale for manipulating microenvironmental conditions and the expression of key photosynthetic genes, which may help growers or breeders to optimize development, composition, yield or other economically important fruit quality aspects.

## 1. Introduction

Fruits are important products derived from agriculture and their consumption is indispensable for the human diet. In addition to vitamins, minerals and fibers, several phytochemicals present in fruits are generally regarded as beneficial to human health, by reducing the risk of a wide range of cancers, cardiovascular diseases and other diet-related diseases, possibly through their antioxidant capacity [1]. Due to the high nutritional and economic value of fruits—a perishable staple—many biochemical, physiological and molecular studies have been performed, e.g., to improve their nutritional quality and shelf life [2].

Evolutionary pressures have resulted in a wide diversity of fruits, ranging from small dry seed capsules that burst to allow seed dispersal, to relatively large complex fleshy fruits that have evolved bright colors and complex aromas to attract seed-dispersing birds and other animals [3]. This diversity can be organized under the following dichotomies: fleshy or dry (with/without a soft succulent pericarp) with the dry fruits being either dehiscent or indehiscent (open/not open to discharge seeds). For instance, capsules, siliques and legumes are dehiscent and dry; achenes, nuts and caryopsis of cereal grains are indehiscent and dry; drupes, apples and berries are indehiscent and fleshy. With regard to fruit ripening, the classification of climacteric or non-climacteric fruits is based on their ethylene production and respiration rate [4]. The onset of ripening in climacteric fruits is characterized by an increase in respiration with a simultaneous and well-characterized peak in ethylene production (e.g., apple—[5]; tomato—[6]), while in non-climacteric fruits the ripening process occurs more gradually without sudden changes (e.g., strawberry—[7]; grapes—[8]).

Photosynthesis essentially depends on irradiance, resistance to CO_2_ diffusion (from the atmosphere to the sites of assimilation in the chloroplasts) and surface/depth containing photosynthetic pigments. In plants, photosynthesis predominantly occurs in green leaves, which are the primary sources of photoassimilates to the whole plant. However, it has been demonstrated that throughout the life cycle of the plant also other vegetative and reproductive structures can be photosynthetically active, including green fruits, green stems, green flower organs and even roots (as reviewed by Aschan and Pfanz [9], and more recently by Brazel and Ó’Maoileídigh [10] and by Simkin et al. [11]) (Table 1). The photosynthetic activity of these organs and structures may be seasonal, occurring at specific developmental stages, and may vary between tissues within these structures. For instance, in grape berries at the green phase, a clear tissue-specific distribution of photosynthetic competence was observed by Breia et al. [12]: the exocarp revealed the highest photosynthetic capacity, whereas the mesocarp exhibited very low fluorescence signals and photochemical competence, and the seed outer integument revealed a photosynthetic ability similar to that of the exocarp.

Early studies on fruit photosynthesis in climacteric apples and non-climacteric grapes (both fleshy fruits), indehiscent fruits (e.g., cereal grain) and dehiscent fruits (e.g., pea pod) were reviewed by Blanke and Lenz [27]. They reported that the photosynthetic profile of each type of fruit was dependent on some morphological and anatomical characteristics, as, for instance, pea pods have much less resistance to CO_2_ diffusion than fleshy fruits [27]. Only a few studies have been performed on this research topic, often using different experimental approaches. In addition, the relevant information is dispersed in the literature and should be compiled for a better understanding of the drivers and constraints for fruit photosynthesis to occur. Therefore, this review aims to compile and integrate information focusing on the characteristics of fruits, including anatomical, physiological and biochemical properties, as well as raising discussion on the possible functions of photosynthesis of fruits on their metabolism and development. Additionally, it also highlights several physiological, biochemical and molecular aspects of the photosynthetic active grape berry tissues, since recent studies brought new results and insights related to this research topic [12,28,29,30,31,32,33].

## 2. Anatomical and Physiological Characteristics of Fruits

### 2.1. Cuticular Structure

Fleshy fruits are covered by an outer epidermis coated with a cuticle of variable thickness, which is composed of cutin and impregnated with waxy or greasy layers [34]. During the development of fleshy fruits, the biosynthesis of cuticular wax is regulated by environmental factors such as drought/humidity, light, temperature and pathogens (as reviewed by Trivedi et al. [35]). Like in leaves, in fruits, the cuticle provides a waterproof barrier between the epidermal cells and their relatively dry environment [36]. In addition, during fruit growth and development, the cuticle maintains its integrity with the increasing fruit volume and also plays a central role in protecting the fruit against biotic stresses (e.g., insects and fungi) and abiotic stresses (e.g., UV radiation) [34].

Several studies have been carried out with the aim of understanding cuticle formation in fruits, including apple [37], grapes [38] and tomato [39]. In general, during fruit growth, there is a rapid accumulation of wax in the cuticle, which makes it thicker and hinders the diffusion of gases [27]. For instance, the cuticle of Riesling grape berries is present since their earliest development, with an increasing rate of cutin and wax deposition at the pre-veraison stage, while at later stages, along with the very rapid expansion of fruit surface area, the cuticular material flattens out [40]. In oranges at later developmental stages, several genes involved in the biosynthesis of wax, cutin and lignin are significantly expressed, while genes involved in photosynthesis are repressed [41]. In pepper, the removal of the cuticle increased the gas permeability of the fruit surface [42], suggesting that the fruit cuticle is crucial in determining compositional gradients between the external and internal atmosphere. 

The pods of pea (*Pisum sativum* L.) have two distinct photosynthetic layers, i.e., the outer (exocarp) and the inner (endocarp) epidermis, containing a thick and thin cuticle, respectively. The exocarp controls the diffusion of CO_2_ from the outside atmosphere through the stomata, while the endocarp is involved in the photoassimilation of CO_2_ diffusing from the inner fruit cavity and mainly resulting from seeds respiration processes [43].

### 2.2. Stomata Density and Functionality

Although stomata are present in the outer epidermal layers of fruits, their density is 10 to 100 times lower than in the abaxial epidermis of the respective plant leaves [9]. Despite this, in young fruits, the stomata are as sensitive to gases as in leaves and regulate the rate of CO_2_ exchange. However, upon growing the fruit surface expands and the density of stomata decreases, and in full-grown fruits, the diffusion resistance to CO_2_ is mainly determined by the lenticels, which are small, round or elliptical, pore-like structures derived from nonfunctional stomata [27]. Table 2 shows the stomatal density of some fruits at a specific developmental stage. 

For instance, in grape berries, the stomata are functional until veraison, while after that stage the stomata density decreases to less than one per mm^−2^ [44] and they become nonfunctional (turning into lenticels covered by wax). These developmentally induced changes result in a decrease in tissue transpiration and water loss, but also in higher CO_2_ and lower O_2_ concentrations inside the berry [50]. In cherry fruit the stomata are not uniformly distributed over the surface of the epicarp: instead, they are found in high numbers near the apex and only a few are present near the stem end [51,52]. In cucumber fruit, the stomatal frequency is 12.5 mm^−2^ which is only 1.58% and 0.91% of the upper and lower surfaces of leaves, respectively [13]. The stomata of a ripe apple are 30 times less abundant than on the abaxial surface of the respective leaf [27]. In young peach fruits, the stomata density is high, leading to a high conductance, while at maturity the stomata lose their function and differentiate into lenticels [53]. Additionally, in chili peppers, stomata are absent in the inner and outer epidermis of mature fruit, and gas exchange can only occur through the cuticle [54]. Scanning electron micrography of mature olive fruits demonstrated that the stomata are covered by a wax layer of complex architecture [15].

Stomata were also observed in the external surface of pods, but again at lower numbers as compared to leaves of the same plant. For instance, in chickpeas, the external pod surface has a mean stomatal density of 31 ± 3 mm^−2^ compared to 126 ± 6 mm^−2^ in leaves [48]. The same study demonstrated a higher rate of transpiration and a lower water use efficiency in old pods as compared to young pods, due to an increase in leakiness of the stomata with pod aging. The inner epidermis of the endocarp of pea pods has thin cuticles lacking stomata, while the outer epidermis, with thick cuticles, contains stomata at a density of approximately 25% of that of the leaflet, suggesting its importance for the atmospheric CO_2_ uptake [43] and regulation of water loss in the pods. In soybean pods, functional stomata are present at the early stages of growth, thus enabling gas exchange [55]. Similarly, the stomata of *Brassica* pods are more functional at early developmental stages compared to later phases, with the stomatal conductance reaching a maximum at 30 days after anthesis [56].

The grains of cereals have a green pericarp, in which stomata are occasionally present on the external surface. In fact, there are contradictory data concerning this issue. Cochrane and Duffus reported very few stomata in wheat pericarp, which are probably insufficient for effective gas exchange with the outside environment [57]. On the other hand, Tambussi et al. [58] did not find any stomata in the wheat pericarp, whereas high stomatal densities were observed in the awns, glumes and lemmas. These latter authors suggested that the photosynthetic activity of the green pericarp is dependent on CO_2_ internally generated by the respiration of the endosperm cells.

### 2.3. Light Diffusion inside the Fruits

The morphology and anatomy of fleshy fruits (e.g., large volumetry) impose physical constraints to light penetration into the inner tissues, eventually reducing the active photosynthetic zone to the outermost layers [12]. In addition to its intensity, also the quality of light reaching the inner regions is influenced by the cells of the outer pericarp. The presence of chlorophyll in green fruit strongly influences the spectral composition of the light filtered through the fruit pericarp, as reviewed by Llorente et al. [59].

The photon flux density (PFD) transmission through the fleshy fruit skin ranges from 1 to 47% of the incident PFD and, generally, only 2% reaches the internal regions [9]. For instance, the exocarp of grape berry transmits 47.1% of the incident PFD and that of apple cv. Golden Delicious at about 31.4% [9]. In *Citrus unshiu* the photosynthesis was greater in fruits than in leaves under considerably low PFD values (i.e., 13.5 to 68 µmol m^−2^ s^−1^), suggesting that fruit photosynthesis resembles that of shade plants [45]. Green peel avocado fruit transmitted only 1.5% of the incident light at 660 nm and 8.4% at 730 nm [47], consistent with the spectral light absorbance characteristics of the chlorophylls present in the peel. In inner tomato tissues, such as the locules, a high expression of genes associated with photosynthesis can be observed [60]. Under a constant photosynthetically active radiation (PAR) of 1750 µmol m^−2^ s^−1^, the peel of young apple fruit transmitted 1–3% of incident PAR at 400 nm, increasing to 10–12% at 850 nm [61], and with fruit development, this light transmission into the fruit core decreased by up to 80%. Similarly, in olive fruits, the penetration of PAR into the internal layers decreases upon development due to the increase in fruit volume [15]. Chen and Cheng observed that the sun-exposed peel of apple fruit had a higher photosynthetic O_2_ evolution capacity, as well as higher activities of enzymes involved in the Calvin–Benson–Bassham (CBB) cycle, as compared to the shaded peel [62]. In the exocarp of cucumber fruit was verified the expression of key genes involved in the photochemical phase, for example, associated with light-harvesting complexes (Lhca) of photosystems I (PSI) and light-harvesting proteins (Lhcb) of photosystems II (PSII) [13]. In the same fruit, the decrease in PAR from 200 to 50 µmol m^−2^ s^−1^ in the surface of cucumber, led to a reduction of photosynthetic rate by 60–65% [63].

In pea pods up to 27 days after anthesis, the exocarp and mesocarp (pericarp) absorbed about 67% of the incident PFD of 2200 µmol m^−2^ s^−1^, while the endocarp received 10% and the remaining 23% was transmitted to the seeds in the pod cavity [43]. At later stages of pea pod development, the decline in chlorophyll content in the outer layers resulted in an increase in PAR reaching both the endocarp and the seeds. In this manner, there is a temporal separation between pods and seeds in their ripening-induced loss of photosynthetic capacity. During day time, with an average of 1000 µmol m^−2^ s^−1^ sunlight, developing soybean embryos receive moderate levels of light (5–30 µmol m^−2^ s^−1^) which influences both the growth and the metabolism of the seed [64]. Similarly, in pods of chickpeas, the light transmission through the seed coat increased with development, leading to greater light utilization by the embryos [65].

In C3 cereals the complex structure of the ear makes it difficult for light to be transmitted to the grain. In fact, the grain is surrounded by the lemma and palea, and it is shaded by the glume, which results in low PFD levels reaching the green pericarp and endosperm [58].

### 2.4. Chloroplasts and Photosynthetic Pigments

Microscopic observations revealed the presence of chloroplasts in different fruits, as reviewed by Blanke and Lenz [27]. The density of chloroplasts in fruit tissues is generally much lower than that in leaves, and thus the photosynthetic rate per unit of area (or per gram of fresh or dry weight) is less as compared to that of leaves [9]. For instance, unripe green strawberries contain 0.2–0.6 mg chlorophyll g^−1^ fresh weight, i.e., seven-fold less than the respective leaves [17]. While in leaves chlorophyll is abundantly present in most cells, in fruits its presence is mostly restricted to specific tissues or cell types (e.g., [12]). For instance, in apple chloroplasts are only found in the hypodermal and inner perivascular tissue [66]. In addition, chloroplasts from different fruit tissues may differ in their structure, composition and function. Resorting to the apple example, the chloroplasts in the hypodermal layers are smaller than those in the inner tissue, exhibit grana throughout fruit development and contain starch granules, thus more resembling those found in leaves, and perform C3-type photosynthesis [66]. On the other hand, chloroplasts from the perivascular tissue are larger and present at smaller numbers per cell as compared to the respective leaf, apparently lack starch grains and are characterized by a C4-type photosynthesis [66]. In the case of cucumber, the chloroplasts appear in the inner walls of fleshy parenchyma cells and contain grana stacks that are 1.7-fold larger than in leaves, while their quantity per unit area is lower [13]. Avocado fruit contains sun-type chloroplasts, as found in leaves with C3 photosynthesis, that retain their structural integrity until harvest; these are comprised of grana, with few thylakoids, of starch and of lower chlorophyll contents than the respective leaves on the basis of surface area [47]. 

In grapes, the outer cell layer (i.e., the pericarp) of the flower ovary contains small plastids with loosely arranged inter-granal lamellae and granal thylakoids and with abundant starch grains; for this reason, they can be regarded as amyloplasts [67]. During the post-anthesis period (i.e., between day 0 and approx. day 42 after anthesis) an increase in chlorophyll content as well as in plastid density within the pericarp tissues is observed, as a result of cell division and enlargement of the fruit [67]. Thereafter, the plastids acquire a larger pleomorphic form, being largely devoid of starch granules until the last stages of ripening while containing large lipid-like globules [67]. In the same study, it was suggested that the plastids of the grape pericarp play a central role in the biosynthesis of isoprenoids, including monoterpenes, and thus in the flavor and aroma of the mature grapes and derived wine. 

Chlorophyll fluorescence measurements show that several fruits species, at least during their development and early ripening, possess a functional photochemical machinery—e.g., mango [68], lemon [69], tomato [70], papaya [71], eggplant [72] and white grape berries [12,28,29]. In fact, the light environment of fruits during their growth and development is a determinant factor affecting fruit photosynthetic rate and the levels of pigments and carbohydrates. For example, it has been demonstrated for mandarin cv. Nules Clementine that fruits grown inside of the canopy had lower pigment concentrations (both chlorophylls and carotenoids) and lower sugar levels as compared to those grown outside of the canopy [73]. Similarly, the environmental light conditions influenced the fluorescence ratio of each photosystem in eggplant fruits [72]. 

Throughout fruit development and maturation, the granal structure of the internal chloroplasts disintegrates and the chlorophyll content decreases as a result of chlorophyllase enzyme activity [74,75]. For instance, in ripening tomatoes, there is a fragmentation of the thylakoid membrane of the green chloroplasts and the formation of colored chromoplasts with new carotenoid-bearing structures [76], as detected by confocal laser scanning microscopy [77]. The decrease in chlorophyll content during ripening is observed in several fruits, including papaya [78], olive [15], tomato [79], mandarin [73], apple [80], coffee [81] and white grape berry [28,29]. 

In berry tissues of the white grape cv. Alvarinho, the chlorophyll content in the exocarp decreased six-fold during ripening, from 120 to 20 μg g^−1^ fresh weight, while in the seed integument, it decreased two-fold, from 80 to 40 μg g^−1^ fresh weight [28]. However, transcriptional analysis of exocarp and seed tissue showed that the *Chlorophyll Synthase* gene (*VvChlSyn*) keeps its relative expression of transcripts in both tissues until later stages of development [31]. Interestingly, a recent work using plastids purified from berry skins of the red grape variety Vinhão, showed an increase in the total chlorophyll content from green to mature berries, suggesting that in this variety the ripening-associated transition from green to red is not associated with a loss of chlorophylls [33]. The authors proposed that the skin of these ripening berries maintains its chloroplasts (with chlorophylls), but the green berry color is masked by the accumulation of anthocyanins [33]. Whether these effects of ripening on chlorophylls in berries also hold true for other white or red grape varieties still needs to be investigated.

During fruit ripening carotenoids and other pigments in fruits can undergo dramatic changes in their content and composition, which are for instance dependent on the specific fruit tissue and maturation stage (for more details see the recent reviews by Kappor et al. [82] and by Simkin et al. [83]). Lado et al. [84] provided a comprehensive overview concerning the main carotenoid profiles in fleshy fruits and the pattern of changes during ripening and the different regulatory levels responsible for the diversity of carotenoid accumulation in fruit tissues. In grape berries, β-carotene and lutein are the predominant carotenoids and there is a steady decline of these compounds after veraison, which appears to be related to chloroplast disappearance and to the formation of carotenoid-derived norisoprenoid volatiles (e.g., β-ionone and β-damascenone) [85,86,87]. These volatiles are important for wine aroma, because of their low olfactory perception threshold [88]. In agreement with this, the total carotenoids content (i.e., the sum values of α-carotene, β-carotene and lutein) in grape exocarp and the seed of the white cv Alvarinho decreased during berry development [29]. Similarly, the expression of genes coding for key enzymes of the carotenoid pathway in exocarp (i.e., phytoene synthase 1, *VvPSY1*; and lycopene epsilon cyclase 1, *VvLECY1*) also decreased throughout berry development [31]. Overall, these results suggest that the carotenoid pathway in both grape berry tissues favors the direction towards α-carotene (i.e., the lutein-epoxide cycle) at the green stage, whereas at veraison and mature stages, it relies on the violaxanthin cycle; in seeds both xanthophyll cycles appear to operate at these later stages of development.

In addition, the light microclimate in the canopy also influences the pigments content of both the exocarp and seed of grape berries: at the green stage of development, tissues from berries growing at a high light microclimate (HL, receiving approx. 150 µmol photons m^−2^ s^−1^ on average) had higher contents of chlorophylls and carotenoids than those from low light microclimate (LL, receiving approx. 50 µmol photons m^−2^ s^−1^ on average) [29]. Similar HL-LL contrasting effects of light microclimate were observed for the photosynthetic activity of both tissues, as determined by in vivo chlorophyll fluorescence [29]. Likewise, the relative expression levels of genes coding for the photosynthesis-related enzymes, such as chlorophyll synthase (*VvChlSyn*) and the expression of some key genes in the carotenoid pathway (e.g., *VvPSY1*; lycopene betacyclase 2, *VvLBCY2*; and *VvLECY1*), were increased by HL microclimate in both tissues, as compared to the LL condition [31]. In particular, HL significantly increased *VvChlSyn* expression in seeds from berries at veraison and mature stages [31], which result may be partly explained by the high dependency of this specific fruit tissue on incoming light, which is consistent with HL seeds being better able to acclimate their photosynthetic capacity to higher light intensities than LL seeds [28]. 

Pea pod walls also have chloroplasts in different cell layers. The outer epidermis of the exocarp contains few chloroplasts, but they are abundant in the inner epidermis of the endocarp (with smaller starch grains), as well as in the parenchyma layers of mesocarp [43]. In the case of chickpea pod walls, similar low levels of chlorophyll were detected in both the embryo and the cotyledons, while the seed coat contained a layer of chloroplast-rich cells directly below the epidermis [65]. The changes in chlorophyll levels occurring in soybean pods are similar to that in fleshy fruits, with a decrease throughout development [55]. These soybean pods contained 9.2–12.4 times less chlorophyll per gram fresh weight than their respective leaves. However, the gross photosynthetic rates on a per mg chlorophyll basis were greater in pods than in leaves, which may be important for the rapid seed-filling period of pods [55].

In cereals, the total chlorophyll content on a dry weight basis is lower in ear parts than in the flag leaf [89]. This difference may be explained by the distinct light microenvironment of these two structures, with the ear localized at the top of the cereal plant and the flag leaf more shaded below. In fact, these authors relate the lower chlorophyll/carotenoid ratio in glumes, lemmas and awns of the wheat ears, as compared to the flag leaf, to their higher need for photoprotection. During the development of the wheat grains, the maximum chlorophyll content in the pericarp green layer was observed 20 days after anthesis, when there was also a peak of photosynthetic activity [90]. 

### 2.5. Assimilation and Refixation of Internal CO_2_

Fruit gas exchange with the external atmosphere takes place mainly through stomata and depends on a diversity of morphological and physiological aspects, namely: fruit type and size, fruit ontogeny stage, fruit temperature, shading and incident PFD, and chlorophyll content [91]. For instance, Aschan and Pfanz [9] mentioned that fleshy fruits perform basically internal CO_2_ refixation, whereas dry fruits, especially during their younger stages, are also able to assimilate atmospheric CO_2_. The incident light also influences the rate of CO_2_ fixation: e.g., in mandarin fruit, the light-saturated net CO_2_ assimilation rate (A_max_) (measured with a portable photosynthesis system using a chamber) was significantly higher in fruits located in the outer canopy than in those in the inner canopy [73]. Overall, fruits assimilate less atmospheric CO_2_ via ribulose-1,5-bisphosphate carboxylase/oxygenase (RuBisCO) when compared to the respective leaves (Blanke and Lenz, 1989). In oranges, as an example, fruits assimilate 50% to 75% less atmospheric CO_2_ than leaves [73]. 

As outlined above, at early development the cuticular and stomata characteristics enable gas exchange in both fleshy and dry fruits. Then, during fruit ontogeny, the great accumulation of wax on its surface leads to a reduction of CO_2_ atmospheric exchange rate (e.g., 10-fold in the case of fleshy fruits) [27] and a simultaneous reduction of water loss via transpiration. Moreover, the increase in mitochondrial respiratory processes, predominantly fueled by imported photoassimilates from leaves at these later stages, results in a rise in the internal CO_2_ concentration [27]. Thus, in the cytosol, the “excess” of internal CO_2_ can be converted by carbonic anhydrase into bicarbonate ions (HCO_3_^−^), which can be subsequently re-fixed by phosphoenolpyruvate carboxylase (PEPC).

In green and red-turning tomato fruit, PEPC enzyme activity is two times higher than RuBisCO activity [70]. Similar results have been obtained for coffee green pericarp, in which the ratio PEPC/RuBisCO activity was higher than in the respective leaves [92]. In the mesocarp of pre-climacteric avocado fruits, the CO_2_ recycling occurs by PEPC, with an enzyme activity around 2.5 µmol CO_2_ s^−1^ g^−1^ fresh weight [47]. In mandarin fruit uncovered with paper bags, the high CO_2_ refixation by PEPC was suggested to contribute to a higher final sugar content at harvest, when compared with fruits covered with bags [93]. More recently, the same authors suggested the presence of a C4/CAM (crassulacean acid metabolism) photosynthetic mechanism in mandarin fruits, based on ^14^CO_2_ labeling experiments [45]. Additionally, by using ^14^CO_2_ incorporation, it was calculated that PEPC enzyme activity in the skin and mesocarp of grape berries was about 17 times higher before veraison than after, leading to a higher concentration of malate and therefore to acidic berries [94]. Further, in apple fruit, PEPC activity was accompanied by malic acid synthesis and a simultaneous regulation of cytoplasmic pH [95], as it is in tomato fruit [96]. More recently, a first quantitative study on in vivo respiratory CO_2_ recapture in non-climacteric fleshy fruits showed that cucumbers, which can assimilate atmospheric CO_2_ via RuBisCO, capture approximately 88% of respiratory CO_2_ via PEPC; this fixation route was quantitatively more important than the direct CO_2_ fixation from the atmosphere [13]. 

Additionally, in the ears of cereals, such as barley and bread/durum wheat, CO_2_ refixation is considered a quantitatively relevant process (as reviewed by Hu et al. [97] and Tambussi et al. [58]). In fact, awns and the external surface of glumes (where stomata are abundant) are the main structures responsible for external CO_2_ assimilation, while the green pericarp and internal surfaces of lemmas (facing the grain), the recycling of respired CO_2_ is the principal process (reviewed by Tambussi et al. [58]). Indeed, it was observed that PEPC plays a role in CO_2_ respiratory refixation, both in barley ears [98] and in chloroplasts of pericarp cells and glumes of durum wheat [99]. Thus, the recycling of respired CO_2_ (i.e., refixation) could have a considerable impact on the final yield of cereal crops, by preventing C loss [97]. 

In legumes, respiratory CO_2_ released from the embryo is re-fixed by a layer of cells on the inner pod wall. In planta, experiments with isotopically labeled CO_2_ (i.e., ^13^CO_2_ injected into the pod space) showed that more than 80% of the label was fixed by the pod walls of chickpea, rather than the seed tissues [65]. This internal recycling of CO_2_ inside the pod may assist in maintaining seed filling in water-stressed chickpea plants [48]. 

## 3. Biochemical Pathways Proposed for Fruit Photosynthesis

**Figure 1:**
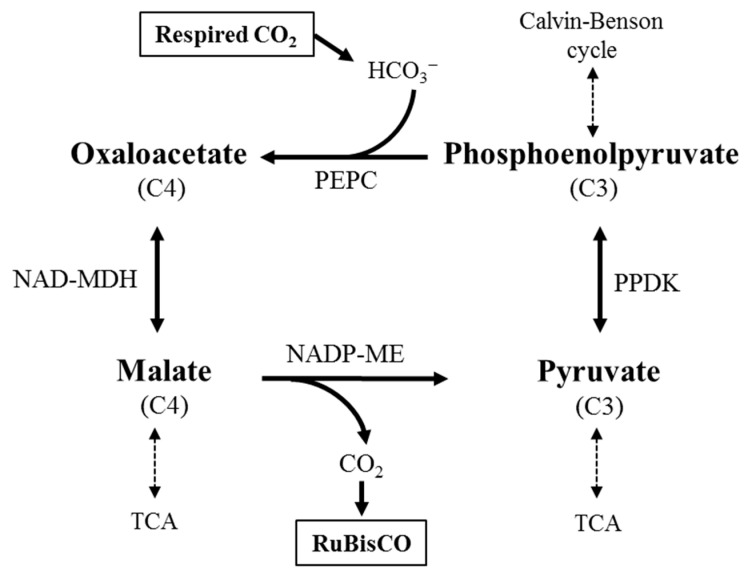
Malate-CO_2_ shuttle proposed by Blanke and Lenz (1989). Abbreviations: HCO_3_^−^, bicarbonate ion; NAD-MDH, NAD-linked malate dehydrogenase; NADP-ME, NADP-linked malic enzyme; PEPC, phosphoenolpyruvate carboxylase; PPDK, pyruvate orthophosphate dikinase; RuBisCO, ribulose-1,5-bisphosphate carboxylase/oxygenase; TCA, tricarboxylic acid cycle. Adapted from Blanke and Lenz (1989) [27]. Reproduced with permission from Michael Blanke, Fruit photosynthesis; published by John Wiley & Sons Ltd., 1989.

More than 30 years ago, Blanke and Lenz [27] proposed a biochemical process for the respiratory CO_2_ refixation, called the malate-CO_2_ shuttle (Figure 1). However, this process could not be generalized for all fruits, as it may vary with the type of fruit, type of tissue within a fruit, the stage of development, etc. Indeed, and based on the available data, Blanke and Lenz suggested that fruits have their own type of photosynthesis, which is an intermediate status between C3 and C4/CAM photosynthesis, rather than categorizing them strictly in C4-type photosynthesis [27].

Here, the excess of respiratory CO_2_ is firstly transformed into HCO_3_^−^ by carbonic anhydrase in the cytosol. Subsequently, a β-carboxylation step of phosphoenolpyruvate (PEP) with the produced HCO_3_^−^ takes place, irreversibly catalyzed by PEPC, to synthesize oxaloacetate (OAA), which in turn is reduced to malate by a cytosolic NAD-dependent malate dehydrogenase (NAD-MDH). Malate can be translocated to the chloroplast and then decarboxylated by NADP-malic enzyme, resulting in CO_2_ and pyruvate; the latter can be regenerated into phosphoenolpyruvate by pyruvate phosphate dikinase (PPDK). The released CO_2_ can be fixated by RuBisCO in the CBB cycle, while pyruvate and malate can be directed to other metabolic pathways, such as the tricarboxylic acid (TCA) cycle and gluconeogenesis or, in the case of malate, accumulated in the vacuole. 

Despite the similarities between this mechanism and C4-type photosynthesis, it is argued that the fruits cannot be cataloged as C4-types, since they do not present Kranz anatomy [27]. However, it is also mentioned that C4 photosynthesis may be also achieved by compartmentalization within the cell [100] without the presence of Kranz anatomy [101]. Additionally, a proxy to CAM mechanism does not imply a spatial separation or Kranz architecture, but a temporal separation of the two carboxylation reactions, eventually associated with the fruit developmental stage. 

**Figure 2:**
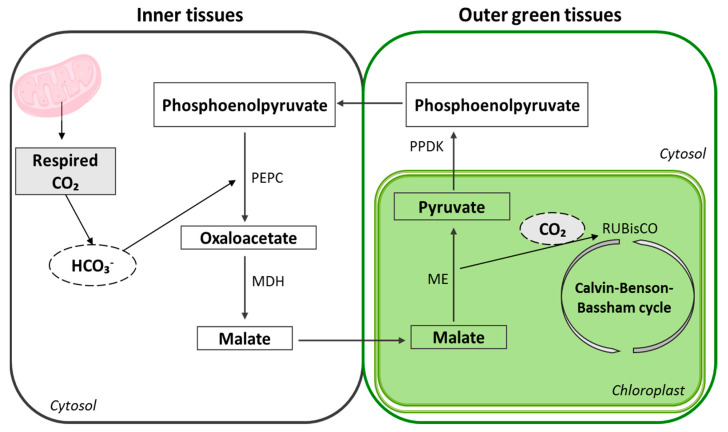
Photosynthesis in non-leaf tissues in outer and inner parts of the organ, proposed by Henry et al. [102]. Key reactions in inner tissues (without light) to capture respired carbon: PEPC, phosphoenolpyruvate carboxylase; MDH, malate dehydrogenase. Reactions in outer tissues (with light): ME, malic enzyme; RuBisCO, Ribulose-1,5-bisphosphate carboxylase/oxygenase; PPDK, Pyruvate orthophosphate dikinase. Adapted from Henry et al. [102]. Reproduced with permission from Robert Henry, Pathways of Photosynthesis in Non-Leaf Tissues; published by MDPI, 2020.

Recently, Henry et al. [102] reviewed the pathways of photosynthesis in non-leaf tissues, including stems, petioles, seeds and fruits. They verified that, in general, the C4 pathway has been reported in these non-leafy tissues in plants that employ C3 photosynthesis in the leaf. Moreover, the authors proposed a process of C4 photosynthesis for those structures, partitioned between the inner shaded tissues (e.g., cells of the endosperm in seeds of wheat), which are characterized by high levels of respiratory CO_2_, and the outer green tissues (e.g., pericarp of wheat) which are more exposed to the light (Figure 2). 

This internal recycling of respired CO_2_ can be an evolutive adaptation of fruits and other non-foliar photosynthetic tissues to avoid carbon losses and thus locally improve their carbon balance [9]. In fact, Henry et al. [102] stated that the main contribution of the photosynthesis in non-leaf tissues seemed to be associated with the need to re-capture carbon, especially in storage organs that have high respiration rates.

For grape berries, Beriashvili and Beriashvili suggested the operation of two main photosynthetic routes, whereby CO_2_ was assimilated primarily as malate in the early stages of development (C4-type photosynthesis, involving PEPC) and subsequently primarily as sugars in ripening berries (C3-type photosynthesis) [103]. This hypothesis is consistent with the strong malic acid vs. sugar accumulation patterns during development and ripening, respectively. The decline in expression of two putative PEPC isogenes and of PEPC enzyme activity from green to veraison stages [104], supports this theory of a ripening-induced photosynthetic switch. In fact, Sweetman et al. [105] suggested that the accumulation of malic acid is in large part due to de novo synthesis in grape berry fruits, through the metabolism of photoassimilates translocated from leaf tissues, as well as those resulting from the photosynthetic activity within the fruit itself. In agreement with this suggestion, proteomic studies revealed that the skin of ripe berries still contains detectable amounts of proteins with functions related to photosynthesis and carbon assimilation [106]. Indeed, both the large subunit and the subunit binding protein alpha of RuBisCO were more abundant in the skin than in the pulp (mesocarp), in accordance with previous studies [107,108]. Thus, the expression pattern of the carbon assimilation proteins indicates that the skin might retain a functional photosynthetic apparatus [106]. In accordance with this, the results of our group using chlorophyll fluorescence analysis demonstrated that grape berry exocarp exhibits a much higher photochemical efficiency than the mesocarp [12], and it keeps this photosynthetic activity until later stages of development [28,29]. In addition, light microclimate also influenced the photosynthesis-related transcripts: at the green stage, HL exocarps showed a significantly higher expression of *VvRuBisCO* (>2-fold) in comparison to LL exocarps [31], in line with the results of their photosynthetic activity [29]. Moreover, the relative expression of *VvRuBisCO* in exocarp increases during berry development, similar to the expression patterns of the *VvSPS1* gene [31], which encodes the sucrose-phosphate synthase enzyme that is involved in sucrose biosynthesis. These results suggest an active photosynthetic/CBB cycle function until late in berry development in both grape berry tissues, and particularly in the exocarp. Thus, from the veraison stage onwards the C-intermediates (e.g., triose-phosphates, erythrose-4-phosphate and 3-phosphoglyceric acid) inside the berry chloroplasts or from the chloroplasts can fuel the biosynthesis of other metabolites.

In apple, a similar malate metabolism and simultaneous recycling of respiratory CO_2_ was verified by Blanke [95]. In fact, the PEPC of apple fruit is a very efficient enzyme with high affinity to its two substrates, HCO_3_^−^ and phosphoenolpyruvate, similar as in CAM or C4 photosynthesis. Here, PEPC gene expression was high only until fruit set, decreasing thereafter during maturation; thus, the further fruit metabolism mostly depends on its initial PEPC protein resource. Malate dehydrogenase was 80-fold in excess in comparison with PEPC, but both were concentrated in the vascular tissue linking the calyx, core and peduncle.

In addition to the reported differences between fruits, it is possible that these mechanisms can occur in the same fruit (spatially or temporally separated) and may or may not be interconnected. In this way, photosynthesis can occur in a typical C3 way (photochemical phase and CBB cycle with CO_2_ fixation by RuBisCO); in a type C4/CAM way, with internal CO_2_ refixation with production of malate, which after being decarboxylated, is fixed by RuBisCO in the CBB cycle; or the CO_2_ fixation being just/mainly internal HCO_3_^−^ refixation with malate production, which can be used for other purposes. All these mechanisms may depend on the tissue, developmental stage, light microclimate, among other factors.

## 4. Possible Roles of Photosynthesis in Fruits

Considering all the anatomical, histological, physiological and biochemical aspects, as well as temporal/developmental effects discussed above, in the present section the possible roles of photosynthesis in fruits will be addressed, as well as the particular case of photosynthesis in seeds.

### 4.1. The Relationship between Photosynthesis and Metabolism in Fruits

The role of photosynthesis in fruit metabolism, and consequently on its quality, has been extensively discussed in tomato [109,110,111,112,113]. The main objective of those works was to obtain answers for the questions: What is the contribution of fruit photosynthesis to fruit metabolism before ripening? Does it have any effect on the final quality of the fruit?

In tomato fruit, genetic and molecular approaches have been applied to understand the relationship between photosynthesis and fruit metabolism, composition and nutritional value. For instance, experiences with transgenic tomato plants, where the expression of chloroplastidial isoform of fructose 1,6-bisphosphatase (cp-FBPase) was inhibited using antisense technology, were performed [109]. The FBPase catalyzes the conversion of fructose 1,6-bisphosphate to fructose 6-phosphate. In green plant tissues there are two isoforms, being the plastidial isoform an important enzyme for the control of CBB cycle. The results showed that the transgenic lines had few changes in their carbohydrate metabolite levels, probably due to its ability to import sugars from leaves. The authors explained this by considering that if there is indeed repression of photosynthesis in the fruits of the transgenic lines, an increased sucrose import becomes necessary as compensation. In addition, the same authors observed that the repression of cp-FBPase enzyme led to a reduction in the average weights of fully ripe fruits. Interestingly, this decrease in weight was quantitatively similar to the estimated contribution of the fruit to the photoassimilates production, that is, around 15–20%, although it represents a minor component when compared to that imported from the leaves [113]. 

In another study in tomato, a downregulation of an auxin response factor (i.e., *ARF4*, which is from a class of transcription factors that regulate auxin-mediated gene expression) resulted in a higher transient starch accumulation at the early stages, together with an enhancement of chlorophyll content and photochemical efficiency, which is consistent with the idea that the photosynthetic activity of fruit may be responsible, at least partially, for the production of photoassimilates and, consequently, for the high levels of starch [112]. In addition, it was verified that the overexpression of the transcription factor *SlGLK2* (important for chlorophyll accumulation and distribution in developing fruit), led to an enhancement in the expression of genes associated with fruit photosynthesis and chloroplast development, and also to an increase in carbohydrates and carotenoids in ripe tomato fruit [111]. These studies in tomato point to a contribution of photosynthesis in supplying assimilates, such as sugars/starch and other metabolites needed for the growth of the fruit (e.g., lipids) at the early stages of fruit development, whereas at later stages this photosynthesis may supply carbon sources to other biosynthetic pathways, such as carotenoids or compounds important for the final quality of the fruit. 

Moreover, these studies also pointed to the importance of fruit photosynthesis to carbon use efficiency, and its implications on fruit yield and quality, as recently reviewed by Simkin et al. [11]. Ultimately, manipulation of key processes to increase fruit photosynthesis could provide a novel target for breeding programs or genetic engineering, with the prime objective to improve fruit and cereal crop yield, quality and nutritional value, as reviewed by Simkin et al. [11,114,115]. For instance, transgenic wheat plants with increased activity of the CBB cycle enzyme sedoheptulose-1,7-bisphosphatase (SBPase), revealed increased gross photosynthesis in their ears relative to the wild-type control [116]. Similarly, in tomatoes, an increase in SBPase activity results in a significant (>30%) increase in plant biomass [117].

The contribution of fruit photosynthesis to the overall carbon budget (as shown in Table 3), was assessed by: (1) calculations using CO_2_ gas exchange measurements, dry weight and carbon content (e.g., [118]); (2) ^14^CO_2_ feeding and gas exchange [13]; or (3) by calculations using electron transport rates, determined by pulse amplitude modulated (PAM) fluorometry, and surface area [119]. However, it is important to be aware of the challenges and methodological limitations associated with measuring photosynthesis in non-foliar tissues, as reviewed by Lawson and Milliken [120] and by Simkin et al. [11]. For instance, the gas exchange technique can underestimate photosynthetic rate, due to the two possible sources of CO_2_, the atmosphere and the mitochondrial respiration (or other internal sources). Alternatively, combining thermography and other approaches, such as chlorophyll fluorescence, can provide insights into spatial and temporal stomatal behavior, which may be valuable to elucidate and quantify atmospheric CO_2_ fixation from other CO_2_ sources [120]. 

However, this is only about carbon, and photosynthesis can provide more than carbohydrates or even C-skeletons. Therefore, understanding the various functions of fruit photosynthesis is crucial, since it can provide a potential route for manipulating key photosynthetic genes to enhance fruit development, composition, yield or nutritional quality, particularly under conditions of stress when leaf photosynthesis may be compromised. In that regard, reviews concerning the photosynthetic characteristics of non-foliar organs in C3 cereals concluded that overall, the non-foliar organs (e.g., the ears), had higher tolerance to environmental stresses (e.g., water stress), as compared with the leaves [58,97]. A possible explanation for this difference is that water relations in these organs are considerably different to foliar tissue and stomata could play a key role in this regulation (as reviewed by Tambussi et al. [58] and Lawson and Milliken [120]). In fact, stomata have been found on both the adaxial and abaxial sides of glumes and lemma of the ears [11], and therefore, the stomata can support both atmospheric CO_2_ uptake from one side of the tissues and refixation of respiratory CO_2_ uptake on the other side [120]. Thus, the capacity of photosynthetic components under drought (delayed senescence), might help the ear to continue to fix CO_2_ late in the grain-filling period [58,97]. Still concerning the cereal crops, recently, Araus et al. [124] and Sanchez-Bragado et al. [125] reviewed the different strategies to increase the photosynthetic rates and the impact of these strategies in terms of increasing biomass and/or yield of non-laminar organs. The authors presented a summary of the net photosynthesis rate in different green photosynthetic organs of C3 cereals. For instance, they observed that the ear photosynthesis per unit area is, in general, lower than flag leaf photosynthesis, but, when expressed per whole organ, ear photosynthetic values may be even higher than in the flag leaf and could significantly contribute to total canopy photosynthesis [124].

This theme is complex and still controversial. In fact, Lytovchenko et al. [126] mentioned that tomato fruit photosynthesis is not important for photoassimilate accumulation, including those metabolites impacting taste, and consequently it is not required for fruit metabolism and development. However, the same authors verified that tomato photosynthesis had a considerable role in seed development, impacting on seed set, composition, and morphology during the early developmental stage. In the same way, a recent review about the primary metabolism of apples, suggested that apple photosynthesis may be mainly used to support seed development and accumulate malate via the malate-CO_2_ shuttle [127]. This hypothesis was based on the low rates of CO_2_ assimilation in immature apples, as compared to those of the leaves, meaning that the CO_2_ fixed in the peel also has little effect on growth and development in this crop [128,129].

Regarding grape berry, studies applying transcriptome [104,130,131,132], proteome [106,133,134] and metabolome [130,134,135] analyses, and integration of these data into omics networks to identify putative stage-specific biomarkers [136,137], as well as gene co-expression networks [138], identified components directly related to photosynthetic activity before veraison, namely light harvesting complexes, the photosystem II oxygen evolving complex and CBB cycle enzymes. In particular, the downregulation of photosynthetic genes after veraison was first analyzed by expressed sequence tag (EST) profiling [139,140] and confirmed by microarray analysis in grape berries [104,137,141,142], and specifically in berry skins [143], wherein photosynthesis-related transcripts are more abundant [131]. Overall, these results are in line with our data obtained by chlorophyll fluorescence in the grape tissue exocarp and seed [12,28,29]. Interestingly, a recent proteomic work using the red grape berry skins (cv. Vinhão), showed that several proteins of the light reactions (e.g., antenna complex, PSII, PSI, cytochrome *b6/f*, and ATP synthase) were significantly accumulated in the chloroplasts at the mature stage as compared to green stage [33]. Moreover, the transition from the green to mature stage was accompanied by a strong decrease in proteins involved in the biosynthetic reactions of the CBB cycle [33]. The authors suggest that at the green stage the CBB cycle can provide carbon compounds to the biosynthesis of amino acids, whereas at the mature stage the chloroplasts may provide adenosine triphosphate (ATP) for cell maintenance and metabolism (energy-demanding processes) or even O_2_ to feed the respiratory demand of inner tissues [33]. Clearly more studies, preferably with different grape varieties, are needed to conform these proteomic results and draw general conclusions.

Indeed, the CBB cycle can supply precursors for pathways of primary and secondary metabolism (as reviewed for tomato by Cocaliadis et al. [113]; and for apple by Tijero et al. [127], who highlighted the importance of apple photosynthesis for its primary metabolism). Primary metabolites, such as sugars, organic acids, and amino acids, produced at the early stages of development, determine most of the final fruit quality, as they are the main substrate for many reactions of secondary metabolism. Secondary metabolites, such as phenolic compounds, play important roles in plant defense against biotic and abiotic factors [144]. Additionally, the diversity of compounds contributes for the nutritional quality of fruits and for its organoleptic properties, which are important to make them attractive and palatable. For instance, in grape berries, phenolics contribute to the color, taste, texture and astringency of the wine, as well as to its antioxidant properties and beneficial effects on health [145,146]. These compounds are mainly present in the exocarp and seeds of the grape berries [30,147], both photosynthetically active [12,28,29].

In particular, large-scale untargeted metabolomics analysis showed that the exocarp from the green stage had relative high levels of flavan-3-ols and of stilbenes, including resveratrol, when compared to the mature stage [30]. Similarly, the expression of genes from the phenylpropanoid (i.e., phenylalanine ammonia lyase 1, *VvPAL1*) and stilbenoid (i.e., stilbene synthase 1, *VvSTS1*) pathways, as well as of those associated with flavan-3-ols biosynthesis (i.e., dihydroflavonol reductase, *VvDFR*; leucoanthocyanidin reductase 2, *VvLAR2*; and anthocyanidin reductase, *VvANR*), was also higher at the green stage than in subsequent stages [31]. Moreover, the exocarp showed an increase in the abundance of several flavonol glycosides along berry ripening [30], which was consistent with the expression of flavanol synthase 1 gene (*VvFLS1*) [31]. Thus, the CBB cycle in the exocarp, which is still active until later stages of berry development (c.f. *VvRuBisCO* expression) can contribute with carbon skeletons needed in the flavonoid pathway. However, further studies are required to better understand this possible crosstalk/relationship between photosynthesis/photochemistry/CBB cycle and secondary metabolism in grape berry.

### 4.2. The Particular Case of Photosynthesis in Seeds

In addition to fruit tissues, some seeds are green (chlorophyll-containing) during part of their development (e.g., grape berry seeds—[28]; immature coffee grains—[148]), or even until germination [149]. During maturation, the seeds lose their green color due to a decrease in chlorophylls and carotenoids, as seen for grape berry seeds [29] and coffee grains [148]. Although the machinery and the mechanism of the photosynthesis of leaves and green seeds seem to be quite similar, there are differences in characteristic features between the leaf and the green seed photosynthesis in higher plants, as recently revised by [149]. For instance, as compared to the leaves, the photosynthetic green seeds have: low chlorophyll *a* and *b* content; high chlorophyll *a* to *b* ratio; chloroplast with broad granal stacks; abundant PSII associated to proteins; higher PSI activity than PSII activity; and high potential to harvest at low PFD [149].

Research has been conducted to understand the specific physiological functions of photosynthesis in these organs [64,149,150,151,152,153]. In general, seed photosynthesis may contribute in three distinct ways: (1) supply of oxygen to prevent and/or reduce hypoxia and hence to aid the high respiration rates during seed germination; (2) production of nicotinamide adenine dinucleotide phosphate (NADPH) and adenosine triphosphate (ATP), both fundamental for energetically demanding biosynthetic pathways in the chloroplast such as fatty acid synthesis; (3) provision of C-intermediates for primary and secondary metabolism by the CBB cycle; and (4) re-fixation of respiratory CO_2_ by RuBisCO, which process can improve the energy efficiency of seeds. 

#### 4.2.1. Possible Functions of O_2_, ATP and NADPH from the Photochemical Phase

Most seeds have some peculiarities that hinder the absorption of oxygen, such as a thick coat and the accumulation of proteins and oils that become a glassy matrix during desiccation [154], or being located deep in dense mesocarps as in some fleshy fruits as the grape berry [12]. Thus, during the maturation and desiccation process, there is a decrease in oxygen diffusion into the dense inner seed tissues. This situation of hypoxia causes restrictions to the production of ATP by mitochondria (oxidative phosphorylation), which is pivotal for various metabolic pathways during seed development and embryo maturation [155,156,157]. Hypoxia can directly or indirectly affect several other processes in seeds, such as nutrient uptake (e.g., wheat—[155]), storage activity and metabolite distribution (e.g., soybean—[151]), assimilate partitioning between endosperm and embryo (e.g., maize—[158]), and enzymatic activities associated with lipid metabolism (e.g., rapeseed—[157]). It is appealing to assume seed photosynthesis to the production of O_2_ in the photochemical phase thus avoiding hypoxia conditions inside of seeds.

Monocotyledonous barley caryopsis (*Hordeum vulgare* L.) has a green pericarp with chlorophyll (called chlorenchyma), where photosynthesis occurs in the mid-storage stage [159]. Chlorophyll fluorescence images of the effective quantum yield of PSII allowed to verify that the photosynthetic activity was restricted to the chlorenchymatous regions of the pericarp, and the photosynthetic activity in these regions was responsible for the production of 3.5 µmol NADPH h^−1^ and 2.3 µmol ATP h^−1^, both important for storage [152]. The oxygen distribution (“oxygen maps”) in barley allows to better understand the photosynthetic activity in its tissues [160]. In general, the pericarp exhibits high levels of oxygen, while in the central regions, there is an oxygen deficiency. In the dark, the oxygen levels decrease dramatically in the inner endosperm region and in the transfer cells (transport pathway of assimilates to endosperm) [161]. On the other hand, in the light, this reduction was not so great, suggesting that the oxygen released by photosynthesis may play an important role in nutrient transport to the endosperm. In addition, the expression of photosynthesis-related genes peaks before the assimilate storage phase [162].

Legume seeds, such as soybean (*Glycine max* (L.) Merril), present an embryo that turns green at the early stages of development. Approximately only 10% of incident light is available to the embryo surface [151]. However, soybean embryos exhibit specialized chloroplasts with high grana stacking. The oxygen map of soybean showed that the seed coat presents high concentrations of oxygen (at the early storage stage), but these values decrease to minimum levels within the liquid endosperm. Furthermore, when measured in the dark, the oxygen concentration within the embryo is much lower (2 µM) comparatively to that under light conditions (220 µM) [151]. At the late-storage stage, soybean embryos have less oxygen concentration and a lower variation (in the same conditions), suggesting a decline in the capacity to balance oxygen consumption with its supply [151]. Pulse amplitude modulated (PAM) fluorescence analysis allowed confirming this situation. In fact, the effective quantum yield of PSII showed a homogeneous pattern for small embryos at the early storage stage, but at the mid-/late storage stage, there was a gradient declining towards the interior of the embryo, suggesting a gradual loss of photosynthetic ability [163].

Studies in grape berries using chlorophyll fluorescence imaging have demonstrated that, in addition to the exocarp, the outer integument of the green seed had a very high fluorescence signal corresponding to high effective quantum efficiencies of PSII [12]. Later on, it was shown that the seed integument had higher values for photochemical efficiency and capacity at green stages of development, similar to those of the exocarp, decreasing at later stages [28,29]. Despite the increase in the volume of the grape berry throughout the development stages, seeds can receive diffuse transmitted light (2.3%), as already reported by Aschan and Pfanz [9], allowing photosynthetic activity at later stages even if at lower levels. Therefore, and based on the light intensity that we previously measured at the grape berry surface level [29], the grape berry seeds received an estimated mean light intensity as low as 1.15 and 3.35 µmol photons m^−2^ s^−1^, at LL and HL microclimate, respectively, which nevertheless support distinct photosynthetic activities in the seeds [29].

This photosynthetic activity of grape seeds can provide the O_2_ necessary to avoid the hypoxia that exists in grape berries [164]. Moreover, oxygen can fuel energy-generating biochemical pathways, including mitochondrial respiration, or others that require the consumption of O_2_ such as lignification [165]. In accordance with this, results from our own studies showed that from the green to veraison stage, seeds had an increase in a lignan-type [30], which is associated with lignin synthesis [165], in agreement with the degree of lignification of grape seeds along development [166].

Additional research in developing seeds (soybean, rapeseed and oilseed rape) suggested that the photosynthetic activity can supply the energy (ATP) and reduction power (NADPH) necessary for lipid biosynthesis, storage metabolism and redox modulation of biosynthetic enzymes [150,151,163,167]. Furthermore, the biosynthesis of fatty acids is a light-dependent reaction using the reducing power and adenosine triphosphate (ATP) generated during the photochemical phase of photosynthesis [168,169]. In this line, studies with legume seeds point to an effect of light on lipid metabolism via seed photosynthesis [64,167] and Ruuska et al. [150] showed that *Brassica napus* seeds in siliques exposed to light in planta produce more fatty acids than seeds from shaded siliques. 

Previously, we showed that in grapes at the green stage, the photosynthetic capacity of HL seed was higher compared to that of LL seed, whereas in the mature stage, the values were similar for both light microclimates [29]. In addition, our metabolomic results showed that the light microclimate influences seed lipid profiles in grapes at all developmental stages: at the green stage, HL seeds had higher levels of ceramides, while at the mature stage, they contained higher relative levels of triacylglycerols and glycerophospholipids, as compared to LL seeds [32]. Thus, at the green stage, seed photosynthesis can supply energy, reducing power and oxygen to fuel the de novo biosynthesis of fatty acids and ceramides. In particular, the HL-induced increase in the relative abundance of ceramides may be associated with seed morphogenesis, cell growth and differentiation that occurs at early developmental stages [170,171]. On the other hand, at the mature stage, the photosynthesis of HL seeds can contribute to the biosynthesis of storage lipids/oils (which are associated with the reserve accumulation), and thus contribute to the final quality of the grape seeds.

#### 4.2.2. Intermediates from Photosynthesis Used in Seed Metabolism and RuBisCO as a CO_2_ Rescue Mechanism

Seeds have high concentrations of internal CO_2_, mainly produced by respiration, but the CO_2_ released during oil synthesis also impacts the overall carbon economy of seed [172]. In addition, the seed coat can be quite thick and hard, making it difficult for CO_2_ to escape. In this sense, the refixation of the respiratory CO_2_ could be an adaptive “strategy” of the green seeds to cope with the cellular acidification caused by this high partial pressure of CO_2_ [150].

**Figure 3:**
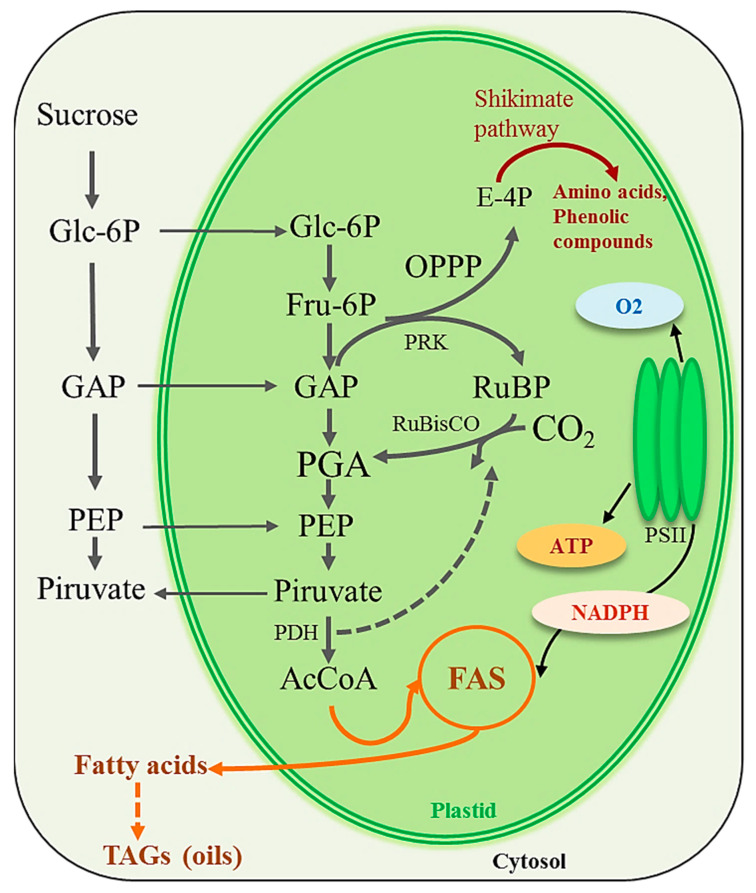
Metabolic pathway of transformation of sugars into fatty acids and highlighting the possible contributes of photosynthesis. Abbreviations: Glc-6-P, glucose-6-phosphate; GAP, glyceraldehydes-3-phosphate; PEP, phosphoenolpyruvate; Fru-6P, fructose-6-phosphate; PGA, 3-phosphoglyceric acid; PDH, pyruvate dehydrogenase; AcCoA, acetyl-CoA; FAS, fatty acid synthesis; OPPP, oxidative pentose phosphate pathway; PRK, phosphoribulokinase; E4P, erythrose-4-phosphate; RuBP, ribulose-1,5-bisphosphate. TAGs, triacylglycerides. Adapted from [64,150,173]. Reproduced with permission from John Ohlrogge, The capacity of green oilseeds to utilize photosynthesis to drive biosynthetic processes, 2004, 136, page 2706, and by permission of Oxford University Press.

Furthermore, during embryogenesis, seeds receive photoassimilates from the phloem that are used for growth and for the synthesis of reserves, being this metabolic pathway characterized by the conversion of sucrose to pyruvate, through glycolysis, which is then transformed by pyruvate dehydrogenase (PDH) in acetyl-CoA. This is the main precursor of biosynthesis of fatty acids, which are then used towards triacylglycerides or triacylglycerols synthesis (TAG, or storage lipids or oils) [173]. This conversion of sugars results in the loss of carbon, in the form of CO_2_, for each acetyl-CoA unit produced. According to Ruuska et al. [150], RuBisCO provides another route for the fixation of CO_2_ released by the PDH. Corroborating this view, from a study with embryos of *Brassica napus* L. (oilseed rape), Schwender et al. [173] described a new metabolic pathway, in which RuBisCO acts without the CBB cycle, in a mechanism previously unknown (Figure 3). 

This metabolic pathway involves three main steps. The first is the conversion of hexose- and triose-phosphates to ribulose-1,5-bisphosphate by the non-oxidative reactions of the oxidative pentose phosphate pathway (OPPP) together with phosphoribulokinase (PRK); the second, the conversion of RuBP and CO_2_ (most of which is produced by pyruvate dehydrogenase—PDH) to 3-phosphoglyceric acid (PGA) by RuBisCO; and third, the metabolism of PGA to pyruvate and acetyl-CoA, and then to fatty acids. Alternatively, the carboxylation of RuBP by CO_2_ released by other internal pathways could also keep the CBB cycle active in non-foliar tissues, mostly in case of a short supply of sugars from leaves with reduced photosynthetic activity (e.g., under abiotic stresses, such as water stress). 

Therefore, both mechanisms would avoid loss of carbon by recycling internal CO_2_, as well as provide several intermediates of the CBB cycle for distinct pathways of metabolism. In fact, intermediates of OPPP pathway or RuBP regeneration phase of CBB cycle, as erythrose-4-phosphate, together with PEP can be used for amino acid synthesis, which in turn can contribute as precursors for the shikimate pathway. This biosynthetic pathway is responsible for the production of phenylalanine, as well as other aromatic amino acids, such as tyrosine and tryptophan. Phenylalanine in the first substrate of a key secondary metabolic pathway, the phenylpropanoid pathway [174]. In this way, seed photosynthesis is essential to provide the nutrients for the growing embryo, such as storage compounds such as starch and oil bodies, rather than producing transportable carbohydrates as is in leaf photosynthesis [149]. In line with this, Schwender et al. [173] showed that this new pathway provides 20% more acetyl-CoA for fatty acid synthesis and resulted in 40% less loss of carbon as CO_2_, comparatively to glycolysis. Similarly, Allen et al. [64] verified that in soybean embryos, RuBisCO re-fixed 11% of the CO_2_ released by lipid synthesis and the TCA cycle, and consequently, the CBB cycle contributed to the carbon economy. 

In the grape berry seeds, despite the decrease in the photosynthetic activity at the mature stage [29], our recent transcriptional data showed that the relative expression of *VvRuBisCO* was maintained in high levels throughout the three developmental stages of seeds, in values similar to those found in the exocarps, and it was also up-regulated by HL microclimate [31]. Therefore, this may suggest that the CBB cycle can be active, until later stages of seed development, especially in HL seeds, as a CO_2_ rescue mechanism, as proposed by Ruuska et al. [150] and Schwender et al. [173]. Thus, the CBB cycle may indirectly provide acetyl-CoA needed for de novo synthesis of fatty acids in the chloroplast and hence fueling the synthesis of triacylglycerols, sterols and glycerophospholipids in these mature seeds [32]. Additionally, the CO_2_ rescue mechanism can provide other intermediates for primary and secondary metabolism. For instance, it can be important to support the high relative abundance of procyanidins at the green stage and of viniferins at the veraison and mature stages [30].

### 4.3. Photosynthesis and the Vascular System of Fruits

Until the later stages of development, sugars are transported from source organs to the fruits, through the phloem. The unloading of these sugars can occur by symplastic or apoplastic pathways, the latter a mechanism dependent on energy [175]. The photochemical phase of photosynthesis can provide the energy (ATP) necessary for the apoplastic unloading [176], an energetically demanding process.

Hibberd and Quick [177] verified that the cells surrounding the peripheral vascular system of stems and petioles of tobacco have chlorophyll and photosynthetic activity (C4-type). Similar results were observed in young shoots and chlorenchyma of lignified shoots of grapevine [24]. Although it was refereed that in grape berries the unloading is predominantly symplastic in the early stages of development, becoming the apoplastic pathway dominant with the onset of ripening [178], our previous work in white grape berry tissues using the chlorophyll fluorescence imaging technique, showed that there was a high concentration of chlorophyll/photochemical activity in perivascular cells of the peripheral dorsal system [12], what is consistent with an association of the unloading process with photochemistry. Moreover, our transcriptional results pointed to a possible crosstalk between photosynthesis and sucrose unloading and breakdown/carbon usage in the berries. In fact, the relatively high levels of transcripts of genes involved in sucrose catabolism (i.e., *VvSuSy1*, sucrose synthase gene), as suggested by Wang et al. [179], combined with the low levels of sucrose-phosphate synthase gene (*VvSPS1*), in the exocarp of the green stage, may support our hypothesis that photosynthesis contributes with energy to that unloading process [31]. Thus, at the initial stage of development, the main part of the sucrose imported through the dorsal vascular system from the leaves, rather than sucrose produced locally in the berry, is crucial to meet the relatively high demand for carbon and energy that sustain the grape high growth rate [180].

Another interesting putative role for photosynthesis in the vascular bundles was observed in cucumber fruit, where the PEPC is present [13]. The refixation of respiratory CO_2_ by PEPC, followed by the synthesis of organic acids that can accumulate in the vacuole, can provide the turgor pressure necessary for cell expansion and fruit growth [13]. The same was suggested for tomato [70,96]. In grape berry, immunohistochemical studies verified that PEPC is present in the vasculature, in the parenchyma cells of the pericarp and within the developing seeds, leading to the hypothesis that PEPC may play a role in the metabolism of the assimilates after their delivery to the fruit [107], and also into the seed at the appropriate time during its development [181].

### 4.4. Ecological Advantages of Green Fruits and Seeds

The advantages or “services” of fruit photosynthesis can also be examined from an ecological point of view, and we can start this topic by saying that a green-colored fruit, located among green leaves, does not facilitate zoochoric seed dispersal. In fact, fruit color influences their ability to be dispersed by animals, namely birds [182]. In this manner, the ripening process composes the mutualistic relationship between fleshy-fruit plants and seed-disperser animals [183]. In addition to the visibility conditions and the visual aptitude of the receiver, the visual signal detectability is determined by its contrast against the background, that is, the conspicuousness of the signal [182]. Young fruits are usually green, but upon ripening their colors can range from red, blue, yellow and orange to brown or even black. Indeed, chlorophyll degradation is generally accompanied by a conversion of chloroplasts into chromoplasts that progressively accumulate high levels of carotenoids. However, some fruits are green even when they are ripe (“green-ripe” or chlorophyllous), for example, cucumber, kiwi, pea, green pepper and green apple varieties [184].

In nature, the dispersion of seeds of fleshy fruits is commonly performed by animals (zoochory) that follow their visual and olfactory senses [185]. For diurnal seed dispersers such as birds, the visual stimuli are particularly important, whereas many of the nocturnal seed dispersers, such as bats and other mammals, rely to a large extent on olfactory stimuli. “Green-ripe” fruits are less conspicuous than fruits of other colors, and are mostly dispersed by mammals such as bats [184]. In this way, the fruits that maintain green until full maturation tend to be dispersed by a limited variety of frugivores. Cipollini and Levey [184] suggested that the ecological advantage of the “green-ripe” fruits consists in their ability to actively perform photosynthesis, which reduces the plants energy costs for fruit production and may enrich the fruit pulp in nutrient rewards for the frugivores. The same authors showed that at high light levels these green-ripe fruits have a positive carbon balance, while at low light levels their high rates of respiration often result in net CO_2_ losses. The “green-ripe” fruits also produce higher seed and pulp masses, resulting in a quantitatively larger offer for their seed dispersers. In addition, the presence of strong odors in these fruits is also indicative that some secondary metabolic pathways may be still active, eventually fueled by photosynthesis [184]. 

In grape berries the photosynthetic tissues are mainly the exocarp and the outer seed integument [12], which both contain a relative high level of tannins, especially accumulated at green stage, decreasing afterwards [30]. Moreover, the two distinct light microclimates in the canopy (LL and HL) led to differences in the total flavan-3-ols in the exocarp at both green and mature stages [30]. For instance, at the green stage the exocarps of high light (HL) grown berries contained more hydroxy-procyanidin trimers, as compared to low light (LL) ones, whereas at the mature stage the flavan-3-ols monomers (e.g., catechin), dimers (e.g., procyanidin B1) and trimers were lower in HL exocarps. From a sensory standpoint, these compounds are correlated with astringency and bitterness of the wine [186]. In terms of eco-physiological functions, they confer protection against fungal and bacterial pathogens, pest insects and larger herbivores (as reviewed by Barbehenn and Constabel [187]). Together these observations suggest roles for fruit photosynthesis with relevance at an ecological level.

Table 4 summaries the principal and putative functions of photosynthesis in non-foliar organs/tissues, which were described in Section 4. 

## 5. Concluding Remarks and Future Perspectives

Fruits are vital organs in plant sexual reproduction and indispensable foods in our diet. Their quality depends on various physiological and biochemical mechanisms that in the end contribute to the accumulation of a plethora of compounds. The main purpose of this review was to compile and integrate information on anatomical, physiological and biochemical features and constraints of different types of fruits and of their seeds, in order to unveil potential biological functions of the photosynthesis performed by some of their green tissues. A diversity of photosynthetic mechanisms or, in some cases, the utilization of part of “old” photosynthetic routes in new solutions to meet tissue-specific demands or alleviate biochemical pressures is discussed. The biological relevance of photosynthesis in fruits is clearly supported by many findings. Overall, the evidences point to some specific roles and functions, such as: (i) the supply of energy (ATP) and reducing power (NADPH), both produced during the photochemical phase, and which can be important for energy-dependent biochemical processes, such as the unloading of sugars from the vascular system or even the synthesis of fatty acids; (ii) the production of oxygen that can prevent and/or reduce the hypoxia inside of seeds; (iii) the re-fixation of respiratory CO_2_ by RuBisCO in the CBB cycle and/or by PEPC (C4-type photosynthesis); and (iv) supply of the carbon skeletons, derived from the CBB cycle, or RuBisCO action outside the cycle coupled to OPPP, that can fuel pathways of primary and secondary metabolism, and thus contribute for the nutritional and organoleptic properties of fruits, as well as to their adaptive value on an ecological scale. 

Photosynthesis of non-foliar tissues, in particular in fruits, is a fascinating topic studied for over 30 years and from very different perspectives. However, due to the complexity of fruit photosynthesis in space (different tissues, compartments, fruit geometries), in time (variation associated with development) and its dependency on environmental factors, key information is still lacking or conflicting. Well-controlled studies and model systems are needed to better identify cause and effect relationships and to integrate global approaches such as systems biology and omics networks. As an example, in vitro culture of cells from different grape berry tissues with distinct photosynthetic competences may enable studying the link between photosynthetic activity and specific changes at molecular and biochemical levels in a more direct and controlled manner. Moreover, it is also important to understand the complexities of coordination between environmental stresses, and fruit photosynthesis and stress responses, since this may provide key information not only for crop improvement in the context of the ongoing climatic changes, but also to meet the growing demand for high quality food produced in a sustainable and safe manner.

## Figures and Tables

**Table 1:** Net photosynthetic (*P*_N_) rates in different plant species and their structures. *P*_N_ is expressed in µmol CO_2_ m^−2^ s^−1^ with the exception of the case reported for *Vitis vinifera* L.

Species	Structure	*P*_N_ (µmol CO_2_ m^−2^ s^−1^)	References
	**Fruit**		
*Cucumis sativus* L.	Cucumber	2.1–2.4	[13]
*Helleborus viridis* L. agg.	-	0.1	[14]
*Olea europaea* L. (cv. Leccino)	Olive	approx. 9 ^a^	[15]
*Ficus carica* L.	Figs	12.9–18.2	[16]
*Fragaria ananassa* L.	Strawberry	1–4	[17]
	**Floral Parts**		
*Helleborus viridis* L. agg.	Sepals	2.3	[14]
*Lilium* hybrid L. (cv. Enchantment)	Anther	2.3	[18,19]
	Tepals	1.8	
*Spiranthes cernua* L.	Flower	2.5	
	Bud	3.7	[20]
	Inflorescence	0.2	
*Caesalpinia virgata* Torr.	**Stem**	7.8	[21]
*Senna armafa* L.		5.8	
*Prunus persica* L.		0.4–1.0	[22]
*Spartium junceum* L.		approx. 8	[23]
*Vitis vinifera* L.		approx. 0.8 (F_v_/F_m_)	[24]
	**Roots**		
*Sonneratia alba* Sm.	Pneumatophores	0.6	[25]
*Avicennia marina* (Forssk.)		0.2	
*Tecticornia pergranulata* (J.M.Black) K.A.Sheph. and Paul G.Wilson	Aquatic adventitious	0.5	[26]

^a^ Measured as gross photosynthetic rate.

**Table 2:** Stomatal density (number of stomata per mm^2^) of fruits of different species and at a specific developmental stage.

Species	Fruit	Stomatal Density (Number mm^−2^)	Stage of Development	References
*Vitis vinifera* L.	Grape berry	1–2	Green and veraison	[44]
		<1	After veraison	
*Citrus unshiu* Marc.	Satsuma mandarin	200–300	Until 63 DAFB ^a^	[45]
		~30	After 153 DAFB ^a^	
*Cucumis sativa* L.	Cucumber	12.5	9 days after anthesis	[13]
*Ribes nigrum* L.	Black currant	0.18–0.20	Not referred	[46]
*Ribes rubrum* L.	Red currant	0.23–0.32		
*Malus domestica* Borkh.	Apple	10–20	At petal fall	[27]
*Persea americana* Mill.	Avocado	50–75	After anthesis	[47]
		<5	At maturation	
*Fragaria ananassa* Duch.	Strawberries	6	After anthesis	[17]
		1–3	Before red color	
		<1	Red fruit	
*Cicer arietinum* L.	Chickpea external pod wall	31 ± 3	Not referred	[48]
*Pisum sativum* L.	Pea pod	30–35	At anthesis	[49]
		24–26	Fully expanded pod	

^a^ DAFB—days after full bloom.

**Table 3:** Fruit photosynthesis contribution to total carbon (%).

Fruit	Stage of Development	Tissue	Fruit Photosynthesis Contribution to Total Carbon (%)	References
Grape berry	Several	Whole/attached fruit	10	[121]
Tomato	Green (near mature)	Skin	15	[119]
Blueberry	From anthesis through fruit ripening	Whole/attached fruit	15	[118]
Mango	Mature (around 1–2 weeks before ripening)	Skin	1	[68]
Peach	Several: from 24 days after flowering until harvest (in biweekly intervals)	Whole/attached fruit	5–9	[122]
Pea pod	--	--	16–20	[123]
Olive	Several: from full bloom to harvest	Whole fruit	40	[15]
Coffee	--	--	20–30	[92]
Cucumber	Mature (i.e., 9–10 days after anthesis)	Exocarp (i.e., skin)	9.4	[13]

-- Not referred.

**Table 4:** Overview of the principal and putative functions of photosynthesis in non-foliar organs/tissues.

Putative Functions	Organ/Tissue	Species	References
**Re-fixation (recycling) of CO_2_** **contributing to carbon economy**	Mandarin fruit	*Citrus unshiu* Marc.	[93]
	Avocado fruit (mesocarp)	*Persea americana* Mill.	[47]
	Grape berry (skin and mesocarp)	*Vitis vinifera* L.	[94]
	Apple	*Malus domestica* Borkh.	[95]
	Tomato	*Lycopersicon esculentum* Mill.	[96]
	Cucumber fruit	*Cucumis sativa* L.	[13]
	Ears cereals: the green pericarp and internal surfaces of lemmas (facing the grain)	Several species	Reviewed by [58,97]
	Ears of barley and durum wheat	*Hordeum vulgare* L. *Triticum durum* Desf.	[98]
	Durum wheat (pericarp cells and glumes)	*Triticum durum* Desf.	[99]
	Pods of chickpea (pod wall)	*Cicer arietinum* L.	[48,65]
**Supply of oxygen**	Legume seeds	(Several species)	Reviewed by [188]
	Broad bean	*Vicia faba* L.	[189,190]
	Pea seed	*Pisum sativum* L.	
- Supporting needs associated with storage activity and metabolite distribution	Soybean seeds	*Glycine max* L.	[151]
	Barley grains	*Hordeum vulgare* L.	[160]
- Supporting needs associated with resource partitioning between endosperm and embryo	Maize grains	*Zea mays* L.	[158]
- Supporting the increased respiratory rate/energy demand associated with lipid biosynthesis	Soybean seeds	*Glycine max* L.	[163]
**Supply of energy (ATP) and reduction power (NADPH)**	Rapeseed	*Brassica napus* L.	[167]
	Soybean seeds	*Glycine max* L.	[150,151]
	Broad bean	*Vicia faba* L.	[190]
	Pea seed	*Pisum sativum* L.	
- ATP demand of unloading at the vascular system	Grape berry exocarp	*Vitis vinifera* L.	[12,31,107]
**Seed development** (e.g., impact on seed set, composition and morphology)	Tomato seeds	*Solanum lycopersicum* L.	[126]
	Apple seeds	*Malus domestica* Borkh.	[127]
- Seed filling	Soybean pods	*Glycine max* L.	[55,64,150]
**Supply carbon intermediates** to the biosynthesis of/metabolic pathways:			
- Isoprenoids	Grape berry pericarp	*Vitis vinifera* L.	[67]
- Malic acid	Grape berry		Reviewed by [105]
- Amino acids	Red grape berry (at green stage)		[33]
- Flavan-3-ols and of stilbenes	Grape berry exocarp (at green stage)		[30,31]
- Flavonol glycosides	Grape berry exocarp (at later stages)		
- Carbohydrates	Tomato	*Lycopersicon esculentum* L.	[109]
- Starch	Tomato	*Solanum lycopersicum* L.	[112]
- Carbohydrates and carotenoids	Tomate (ripe)		[111]
- Primary metabolism	Apple	*Malus domestica* Borkh.	[127]
- Primary and secondary metabolism	Tomato	*--*	Reviewed by [113]
- Fatty acid synthesis and TCA cycle	Oil rape seed	*Brassica napus* L.	[173]

-- Not referred.

## Data Availability

Not applicable.
